# Prediction of transcription factor bindings sites affected by SNPs located at the osteopontin promoter

**DOI:** 10.1016/j.dib.2017.07.057

**Published:** 2017-08-02

**Authors:** Marco Antonio Briones-Orta, S. Eréndira Avendaño-Vázquez, Diana Ivette Aparicio-Bautista, Jason D. Coombes, Georg F. Weber, Wing-Kin Syn

**Affiliations:** a Regeneration and Repair, Institute of Hepatology, Foundation for Liver Research, London, United Kingdom; b Instituto Nacional de Medicina Genómica, INMEGEN, Periférico Sur 4809, Ciudad de México 14610,. México; c James L. Winkle College of Pharmacy, University of Cincinnati Academic Health Center, Cincinnati, OH, United States; d Division of Gastroenterology and Hepatology, Department of Medicine, Medical University of South Carolina, Charleston, SC, United States; e Section of Gastroenterology, Ralph H Johnson Veteran Affairs Medical Center, Charleston, SC, United States

## Abstract

This data contains information related to the research article entitled “Osteopontin splice variants and polymorphisms in Cancer Progression and Prognosis” [1]. Here, we describe an in silico analysis of transcription factors that could have altered binding to their DNA target sequence as a result of SNPs in the osteopontin gene promoter. We concentrated on SNPs associated with cancer risk and development.

The analysis was performed with PROMO v3.0.2 software which incorporates TRANSFACT v6.4 of. We also present a figure depicting the putative transcription factor binding according to genotype.

**Specifications Table**

**Table:** 

Subject area	*Biology, Molecular Biology*
More specific subject area	*Effect of SNPs in binding of transcription factors for the gene osteopontin*
Type of data	*Table and figure*
How data was acquired	*Software PROMO 3.0.2* (using TRANSFAC v.6.4)
Data format	*Analyzed*
Experimental factors	*SNPs sequences were obtained from NCBI Single Nucleotide Polymorphism Database (dbSNP). PROMO parameters were chosen for human sequences and human sites.*
Experimental features	SNPs located in OPN promoter with an effect in cancer risk and prognosis were analyzed to compare which transcription factors are binding in the variant sequences.
Data source location	
Data accessibility	*The data is available in this article*

**Value of the data**

• These data describe how putative DNA-binding sites for transcriptional factors can be created or interrupted by the changes in sequences generated by SNPs in the promoter of osteopontin.
• Differential binding among SNPs genotypes can potentially explain why these SNPs have been associated with changes in the risk of cancer for a specific population.
• This analysis is an example of how important databases, such as those containing SNP genotypes and the predictive tools for DNA-binding sites for transcriptional factors in a specific sequence, could be used to try to select potential signaling pathways modulating the development of cancer.

## 1 Data

The table provided in this article is a list of the transcription factors predicted to bind a DNA sequence at the SNPs contained in the osteopontin promoter. We analyzed only those SNPs that statistically in a population have been shown to have an effect on cancer risk and prognosis for the carriers. For each SNP we present both sequences. Each analysis contains the rs ID and the nucleotide position in reference to the osteopontin promoter; a schematic representation of the binding of the transcription factor to their target sequence; and an analysis of how similar the binding site is compared to its canonical binding sequence.

## 2 Experimental design, materials and methods

**Fig. 1:**
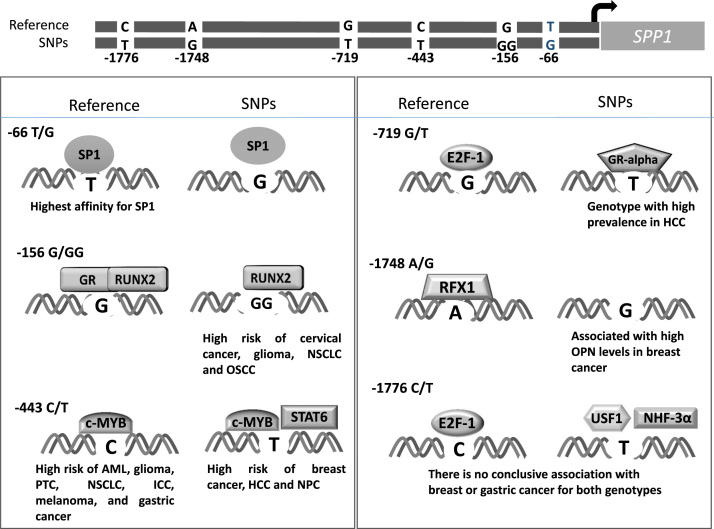
Schematic representation of changes in transcription factors binding to SNPs located in the promoter of the osteopontin gene. At the top of the image there is a representation of the SNPs located in the osteopontin promoter that have been linked to variation in cancer risk in the carriers. The position of each SNP is given with respect to the transcription starting point. Below, for each SNP, the binding of the transcription factors and changes associated with altered genotype are exemplified.

Analysis of SNP sequences was performed using software PROMO v3.0.2, (which utilizes TRANSFAC v6.4) [2,3] For each osteopontin gene promoter SNP, the sequences carrying each allele were loaded as the query sequence to search for potential binding sites. The prediction was carried out considering only sites and only human transcription factors. The output of this analysis is presented in Table 1. Each analysis contains the rs that corresponds to each SNP and its position relative to the transcription start site of osteopontin. For each SNP, we present the respective results for both sequences loaded as the query sequences. A schematic representation (boxes in color, also indicated with numbers) of the binding of the transcription factor to the target sequence, and a list of the putative transcription factors binding to the sequence. For each transcription factor site, several predicted parameters are reported. The *transcription factor name* with the database accession number in brackets; the *start* and *end* positions of the putative binding sequences; *Dissimilarity* (%), which corresponds to the rate of dissimilarity between the putative and consensus sequences for a given transcription factor; *Sequence*, the nucleotide sequence of potential binding site; *Random Expectation (RE)* indicating the expected occurrences of the match in a random sequence of the same length as the query sequence according to the dissimilarity index, presented the *RE equally* (equi-probability for the four nucleotides) and *RE query* (nucleotide frequencies as in the query sequence). Markedly different changes are highlight in grey and the SNP is highlight in red. In Fig. 1 we depict the integration of information obtained from this predictive analysis and data previously reported for transcription factors binding to the osteopontin promoter.

**Table 1:** Transcription factors binding prediction to sequences associated to SNPs genotypes located in the promoter of the osteopontin gene.

## References

[1] M.A. Briones-Orta, S.E. Avendaño-Vázquez, et al., Osteopontin Splice Varinats and Polymorphisms in Cancer Progression and Prognosis. *Biochim Biophys Acta*. **1868**, 2017, 93–108. 10.1016/j.bbcan.2017.02.005 28254527

[2] Farré D., Roset R., Huerta M., Adsuara J.E., Roselló L., Albà M.M., Messeguer X. (2003). Identification of patterns in biological sequences at the ALGGEN server: PROMO and MALGEN. Nucleic Acids Res..

[3] Messeguer X., Escudero R., Farré D., Núñez O., Martínez J., Albà M.M. (2002). PROMO: detection of known transcription regulatory elements using species-tailored searches. Bioinformatics.

